# Chromatin Remodeling Proteins in Epilepsy: Lessons From *CHD2*-Associated Epilepsy

**DOI:** 10.3389/fnmol.2018.00208

**Published:** 2018-06-15

**Authors:** Kay-Marie J. Lamar, Gemma L. Carvill

**Affiliations:** Ken and Ruth Davee Department of Neurology, Northwestern University Feinberg School of Medicine, Chicago, IL, United States

**Keywords:** CHD2, chromatin remodeler, epilepsy genetics, epigenetics, neurodevelopment

## Abstract

The chromodomain helicase DNA-binding (CHD) family of proteins are ATP-dependent chromatin remodelers that contribute to the reorganization of chromatin structure and deposition of histone variants necessary to regulate gene expression. CHD proteins play an important role in neurodevelopment, as pathogenic variants in *CHD1*, *CHD2*, *CHD4*, *CHD7* and *CHD8* have been associated with a range of neurological phenotypes, including autism spectrum disorder (ASD), intellectual disability (ID) and epilepsy. Pathogenic variants in *CHD2* are associated with developmental epileptic encephalopathy (DEE) in humans, however little is known about how these variants contribute to this disorder. Of the nine CHD family members, CHD2 is the only one that leads to a brain-restricted phenotype when disrupted in humans. This suggests that despite being expressed ubiquitously, CHD2 has a unique role in human brain development and function. In this review, we will discuss the phenotypic spectrum of patients with pathogenic variants in *CHD2*, current animal models of CHD2 deficiency, and the role of CHD2 in proliferation, neurogenesis, neuronal differentiation, chromatin remodeling and DNA-repair. We also consider how CHD2 depletion can affect each of these biological mechanisms and how these defects may underpin neurodevelopmental disorders including epilepsy.

## Introduction

Gene regulation is a complex process that is tightly regulated by many factors across tissue types and at different points in development. Chromatin remodelers play an essential role as both activators and repressors of transcription by manipulating the structure of chromatin to allow access for gene regulation machinery or conversely, to obscure sites where gene expression must be repressed. The human nervous system is composed of multiple neuronal cell types, as well as supporting glial cells. These diverse cell types are all derived from neural progenitor cells (NPCs), which then differentiate and migrate to their proper locations during development. Epigenetic mechanisms play an important role in regulating this process and controlling cell fate (reviewed in Borrelli et al., 2008; Yoo and Crabtree, 2009; Hirabayashi and Gotoh, 2010; Riccio, 2010; Luijsterburg et al., 2016). Several classes of chromatin remodelers have been identified, including ISWI, the SWI/SNF family, and the chromodomain helicase DNA-binding (CHD) family of proteins (Hall and Georgel, 2007). In this review, we will discuss the role of the CHD family of chromatin remodeling proteins in neurodevelopmental disorders, focusing specifically on CHD2, which is associated with developmental and epileptic encephalopathy (DEE), a severe form of childhood epilepsy.

## CHD Family of Proteins

Nine CHD proteins have been identified in humans (CHD1–CHD9). The *Drosophila* genome encodes for four CHD proteins (dCHD1, dMi-2, CHD3 and Kismet), while yeast (*Saccharomyces cerevisiae*) only has one such protein (yCHD1; Kahl, 2015). The nine human CHD family members are further divided into subgroups. CHD1 and CHD2 are grouped together due to their DNA binding domain that is not well-conserved in the other CHD proteins (Woodage et al., 1997). CHD3 and CHD4 are grouped together into a second subfamily; these proteins include two plant homeodomain zinc finger domains and function as subunits of the nucleosome remodeling and histone deacetylase (NuRD) complex (Schuster and Stöger, 2002). The third subfamily is more diverse, including the remaining family members CHD5, CHD6, CHD7, CHD8 and CHD9 (Hall and Georgel, 2007). The majority of the CHD family of genes are expressed ubiquitously in human tissues, with only *CHD5* expression being largely confined to neurons (Thompson et al., 2003; Lonsdale et al., 2013).

## The CHD Family and Human Disease

Pathogenic variants in the CHD gene family were first described in *CHD7* in patients with coloboma, heart defects, atresia choanae, growth retardation, genital abnormalities and ear abnormalities (CHARGE) syndrome (Vissers et al., 2004). Since the inception of the next-generation sequencing era, an additional four, *CHD1*/2/4/8, have been implicated in a range of neurodevelopmental disorders (Table 1, O’Roak et al., 2012; Carvill et al., 2013; Weiss et al., 2016; Pilarowski et al., 2017). In contrast to patients with CHARGE syndrome, in whom developmental delay and learning disabilities are rare, the majority of patients with CHD1/2/4/8 pathogenic variants present with intellectual disability (ID) or developmental delay. The severity of intellectual impairment is variable, ranging from developmental delay (*CHD1*), mild to moderate ID (*CHD4*) and mild to profound ID (*CHD2*/*8*). Additional neurological features include epilepsy (*CHD2*) or seizures (*CHD1/8*) and autistic spectrum disorders (ASD; *CHD1/2/8*). Despite the ubiquitous gene expression profiles of all CHD family members implicated in human disease, only *CHD2* pathogenic variants cause a brain-restricted phenotype, suggesting a unique role for this gene in neurodevelopment (Table 1). Alternatively, CHD2 may only have a non-redundant role in the brain, and other CHD family members may be able to compensate for the lack of CHD2 in non-neuronal tissue. This has not been specifically investigated in animal models nor *in vitro* modeling to date. Moreover, given that each of these CHD family members cause distinct phenotypic entities, it is unlikely that the CHD proteins function redundantly across all cell-types.

**Table 1:** Neurodevelopmental disorders associated with germline pathogenic variants in CHD proteins.

Gene	OMIM Gene Number	Inheritance	Proposed pathogenic mechanism	Disorder	Source
*CHD1*	602118	*De novo* (2/5 patients)	Dominant negative	Developmental delay, ASD, speech apraxia, facial dysmorphism	Pilarowski et al. (2017)
*CHD2*/15q26.1 microdeletion	615369	*De novo*	Haploinsufficiency	Myoclonic epilepsy (15q26.1del)	Veredice et al. (2009)
				Generalized epilepsy (15q26.1del)	Dhamija et al. (2011)
				Developmental delay, febrile seizures (15q26.1del)	Li et al. (2008)
				Developmental delay, epilepsy, autistic behavior, facial dysmorphisms (15q26.1del)	Capelli et al. (2012)
				DEE (*CHD2*)	Carvill et al. (2013)
				ID, absence of seizures (*CHD2*)	Verhoeven et al. (2016)
				ASD (*CHD2*)	O’Roak et al. (2014)
*CHD4*	603277	*De novo*	Unknown	ID, macrocephaly, hearing loss, ventriculomegaly, hypogonadism, palatal abnormalities, facial dysmorphism	Weiss et al. (2016)
*CHD7*	608892	*De novo*	Haplo insufficiency	CHARGE syndrome	Vissers et al. (2004)
*CHD8*	610528	*De novo*	Haplo insufficiency	ASD, macrocephaly, facial dysmorphisms, gastrointestinal problems	O’Roak et al. (2012) and Bernier et al. (2014)

*ASD, Autism spectrum disorder; DEE, Developmental and Epileptic Encephalopathy; ID, intellectual disability*.

In addition to *de novo* germline variants, somatic variants in many of these remodelers are associated with various types of cancer, including prostate cancer (*CHD1*), chronic lymphocytic leukemia (*CHD2*), endometrial cancer (*CHD4*) and neuroblastoma (*CHD5*/*CHD9*, Thompson et al., 2003; Liu W. et al., 2012; Rodríguez et al., 2015; Lasorsa et al., 2016). These disease-associations highlight the importance of these remodelers in the maintenance of chromatin structure and the control of cellular proliferation.

## Identification of *CHD2* as an Epilepsy Gene

Several studies found *de novo* microdeletions of chromosome 15 to be associated with epilepsy in single cases using array-comparative genomic hybridization (CGH) and there are a number reported in DECIPHER (Firth et al., 2009). The first published case was a 30-month old girl with developmental delay, seizures starting at 6-months of age, and photosensitivity. This patient carried a 5 Mb microdeletion of 15q26.1–15q26.2 encompassing ~56 genes, including CHD2 (Veredice et al., 2009). Another case study found a 3.3 Mb deletion in the same region of 15q26.1 in a patient with developmental delay and febrile seizures (Li et al., 2008). A third study identified a patient with a much smaller but overlapping deletion, presenting with developmental delay and intractable generalized epilepsy starting at the age of 3.5 years (Dhamija et al., 2011). This deletion is ~0.8 Mb and encompasses only 4 genes, including *CHD2*. Finally, a 511kb deletion at 15q26.1 was found in a patient with developmental delay, epilepsy, autistic behavior, and facial dysmorphisms (Capelli et al., 2012). In this case, the deletion only included two genes: *CHD2* and *RGMA*. Combined, these studies identified the 15q26.1 locus, including *CHD2*, as a potential candidate for severe childhood forms of epilepsy.

With the knowledge gained from these deletion cases, Carvill et al. (2013) included *CHD2* as a candidate gene for epilepsy for targeted resequencing. The group performed sequence analysis on *CHD2* and 64 other known or candidate epilepsy genes in 500 cases of epileptic encephalopathy and found *de novo CHD2* variants in six individuals (1.2% of cases; Carvill et al., 2013). Four of these pathogenic variants lead to truncation of the protein and two are missense variants predicted to disrupt the helicase/ATPase domain. All six patients carrying *CHD2* pathogenic variants presented with myoclonic seizures and varying degrees of intellectual ability, while three of the six exhibited photosensitivity (seizures triggered by flashing lights at certain intensities or certain visual patterns; Carvill et al., 2013).

**Figure 1:**
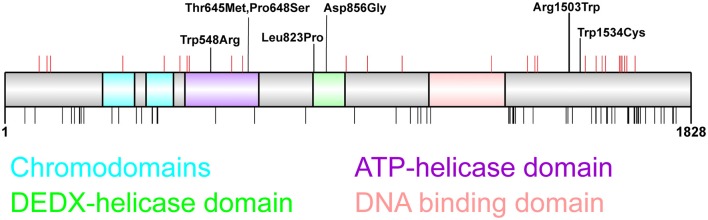
Distribution of chromodomain helicase DNA-binding (*CHD*)*2* pathogenic variants. CHD2 protein schematic showing functional domains and locations of genetic variants. Putative functional domains include two chromodomains, two helicase domains, and a DNA-binding region. The C-terminus of CHD2 also associates with a poly ADP-ribose (PAR) binding domain that is involved in DNA damage repair (Luijsterburg et al., 2016). Pathogenic *CHD2* variants identified in patients with epilepsy and neurodevelopmental disorders (top panel) include truncating variants (red vertical lines denote amino acid position) throughout the protein, and missense variants located in the helicase domains (*n* = 5) and in the C-terminus (*n* = 2). This region of the C-terminus is of unknown function, however the presence of 2 missense variants in patients with developmental epileptic encephalopathy (DEE) suggest this region is important for tertiary structure and/or CHD2 function (Pinto et al., 2010, 2016; Capelli et al., 2012; Neale et al., 2012; Rauch et al., 2012; Carvill et al., 2013; Suls et al., 2013; Chenier et al., 2014; Courage et al., 2014; Hamdan et al., 2014; Lund et al., 2014; O’Roak et al., 2014; Fitzgerald et al., 2015; Galizia et al., 2015; Thomas et al., 2015; Trivisano et al., 2015; Helbig et al., 2016; Lebrun et al., 2017; Wang et al., 2017; Ko et al., 2018; Rim et al., 2018; Zhou et al., 2018). The chromodomains, helicase domains and DNA-binding domain are depleted of missense variation in the general population as indicated by missense variants present more than twice in the GnomAD dataset (black vertical lines, bottom panel; Lek et al., 2016).

Since then *CHD2* pathogenic variants have been identified in a spectrum of patients with early-onset epilepsy (Figure 1). Of the published patients to date, the majority (63%, 25/40) present with developmental and epileptic encephalopathy (DEE). DEE is a group of early onset epilepsy disorders characterized by refractory seizures and cognitive decline or regression associated with ongoing seizure activity (Scheffer et al., 2017). Overall, DEE patients with *CHD2* variants present with seizure onset between 6 months and 4 years, frequently with myoclonic seizures that evolve to multiple refractory seizure types. Clinical photosensitivity is common and some patients will self-induce seizures (Carvill et al., 2015; Thomas et al., 2015). The overwhelming majority of remaining patients without a DEE diagnosis present with ID with epilepsy, and photosensitivity is common. To date, all disease-causing *CHD2* variants in patients with epilepsy arise *de novo*, no transmission (i.e., autosomal dominant inheritance) has been observed, and one mutant allele is sufficient to cause disease. However, there is some evidence that rare variants in *CHD2* could be a risk factor for more common types of photosensitive epilepsies without ID (Galizia et al., 2015). Overall, photosensitivity is a distinguishing feature of this condition, as only ~5% of patients with epilepsy exhibit photosensitivity (Martins da Silva and Leal, 2017).

Pathogenic variants in *CHD2* have also been identified in cohorts of patients diagnosed with other neurodevelopmental disorders, including ASD, ID and developmental delay, some of whom did not present with seizures (Firth et al., 2009; Rauch et al., 2012; Krupp et al., 2017; McRae et al., 2017). O’Roak et al. (2014) identified *de novo* variants in ASD by sequencing 64 candidate genes in 3486 ASD probands and 2493 unaffected siblings using molecular inversion probes. They found that in addition to *CHD8*, which was previously implicated in ASD, *CHD2* was also significantly mutated in ASD. Of the four *CHD2* mutation carriers identified, all have confirmed ASD, and three of the four present with seizures. Another group confirmed the association of *CHD2* with ASD by identifying a missense variant in the helicase domain of *CHD2* in two brothers with ASD, resulting from likely germline mosaicism (Lebrun et al., 2017). In this case, both patients exhibited aggressive behavior and language difficulties, however only one of the brothers presented with seizures. These studies indicate that pathogenic variants in *CHD2* are associated with a range of neurodevelopmental phenotypes, but that epilepsy is the most common neurological feature.

## CHD2: Expression, Protein Domains and Pathogenic Variant Distribution

*CHD2* expression is largely ubiquitous with the highest expression in adult tissue in the thyroid, ovary, lung and the cerebellar hemisphere of the brain (Lonsdale et al., 2013). During human brain development, *CHD2* expression is highest in the neocortex at 9 weeks postconception but remains high throughout development and is expressed postnatally in the brain with highest levels in the cerebellum (Shen et al., 2012). During embryonic development in mice, *Chd2* expression is confined to the developing heart at E10.5, spreads to the forebrain and eye at day E11.5, and appears in the extremities, facial, and dorsal regions by E15.5 (Kulkarni et al., 2008). In adult mice, *Chd2* is expressed in most tissues, with highest expression in thymus, followed by lungs, kidneys, spleen, heart and lower-level expression in testis and liver (brain tissue was not tested for *Chd2* expression in this study; Nagarajan et al., 2009).

CHD2 is a chromatin remodeler that acts as an ATPase to catalyze the assembly of chromatin into periodic nucleosome arrays (Liu et al., 2015). CHD2 is composed of several functional domains, including 2 chromodomains at the N-terminus, an ATPase/helicase domain, and a DNA-binding domain (Figure 1). Experiments using CHD2 deletion constructs identified specific functions of individual CHD2 domains and found that the N-terminus of CHD2, containing the two chromodomains, serves an autoinhibitory role. Deletion of this region increases both DNA-binding and ATPase activities, however this N-terminal region of CHD2 is required for the chromatin remodeling activity of the protein (Liu et al., 2015). The DNA binding domain is able to sense double stranded DNA and enhances the chromatin remodeling activity of CHD2 (Liu et al., 2015).

The importance of each of these protein domains for CHD2 function is illustrated by the presence of missense variants in each protein domain in patients with indistinguishable clinical presentation to patients with truncating variants (Figure 1). These observations highlight the lack of genotype-phenotype correlation between the nature or location of these pathogenic variants and severity or spectrum of clinical presentation. Moreover, it should also be noted that truncating variants do not seem to lead to nonsense mediated decay in patient lymphocytes, and the translation of truncated proteins cannot be excluded (Suls et al., 2013). Overall, the vast majority (83%, 33/40) of patients carry truncating *CHD2* variants, suggesting that the pathogenic mechanism that underpins *CHD2*-associated epilepsy is haploinsufficiency.

## CHD2 Animal Models

*CHD2* is highly conserved among vertebrates; the gene cluster containing *CHD2* (*SLCO3A1-STX-CHD2-RGMA*) has been conserved for over 476 million years, indicated by its presence in divergent species such as opossum, chick and zebrafish (Marx et al., 2007). The human CHD2 protein has high homology to mouse (96.5%) and zebrafish (73.0%), supporting the use of these model organisms to study CHD2 loss (Agarwala et al., 2016).

Knockdown of *chd2* in zebrafish with targeted morpholino (MO) antisense oligomers results in larvae displaying seizure-like behavior and photosensitivity, recapitulating the phenotype seen in humans with pathogenic *CHD2* variants (Suls et al., 2013; Galizia et al., 2015). MO *chd2* knockdown also resulted in several developmental defects such as stunted growth, microcephaly, absent swim bladder, and body curvature in these larvae (Suls et al., 2013). It should be noted that the MO targets a splice donor site, resulting in an abnormally spliced form of Chd2 with a predicted partial deletion of exon 2 that was not characterized as in or out of frame, therefore it is unclear whether this is a full loss of function model. Moreover, a control MO was not used in the photosensitivity analyses (Galizia et al., 2015). Finally, these results should be interpreted with caution, as MOs notoriously cause a range of off-target effects, most notably p53-dependent neural toxicity, wherein 15%–20% of all MOs injected at standard efficacy doses lead to neural cell death due to p53-induced apoptosis (Ekker and Larson, 2001; Robu et al., 2007; Eisen and Smith, 2008; Bill et al., 2009). This phenomenon should be taken into account when attempting to measure and interpret neuronal phenotypes in MO injected animals.

Mice with homozygous deletions of the C-terminus of *Chd2* exhibit perinatal lethality, indicating that *Chd2* is an essential gene for normal development. Heterozygous mice have growth defects and a complex phenotype including cardiomyopathy, glomerulopathy, enlarged spleens, lordokyphosis and gross kidney abnormalities. No neurological defects were reported; however, it is unclear whether a less obvious neurological phenotype may have been missed (Marfella et al., 2006, 2008; Kulkarni et al., 2008; Nagarajan et al., 2009). These results are surprising considering the neurological phenotype seen in both zebrafish and humans with *CHD2* deficiency. This could be explained by the fact that like the zebrafish model, these mice might not be a true model of haploinsufficiency. The *Chd2* mutant mouse was generated through gene trapping, resulting in a CHD2-β-gal-neomycin fusion protein containing the first 1198 amino acids of wild-type (WT) protein, including the chromodomains, helicase domains and N-terminus of the DNA binding domain (Marfella et al., 2007). Moreover, one study showed low level expression of WT *Chd2* mRNA, including the C-terminal region that should be disrupted by reverse transcriptase PCR (Nagarajan et al., 2009). This residual WT CHD2 could interact with mutant CHD2 fusion protein to exert a dominant negative effect. Taken together, it is possible that a dominant negative, or gain of function effect of this CHD2 fusion protein could underlie the discrepancies in affected tissue types in heterozygous knockout mice as compared to humans. Alternatively, the absence of any brain phenotype in these mice could be due to species-specific compensation of CHD2 by other CHD proteins in the mouse brain but not in other tissues. Conditional double knockouts of *Chd2* along with other CHD proteins specifically in the brain, as well as the study of other CHD gene expression in *Chd2* mutant tissues is required to delineate the putative mechanisms that underlie these disparate tissue-specific effects. Moreover, current technologies such as CRISPR allow for more targeted approaches to generating animal models and will be crucial to studying CHD2 function in the future.

## CHD2: Role in Neurogenesis

**Figure 2:**
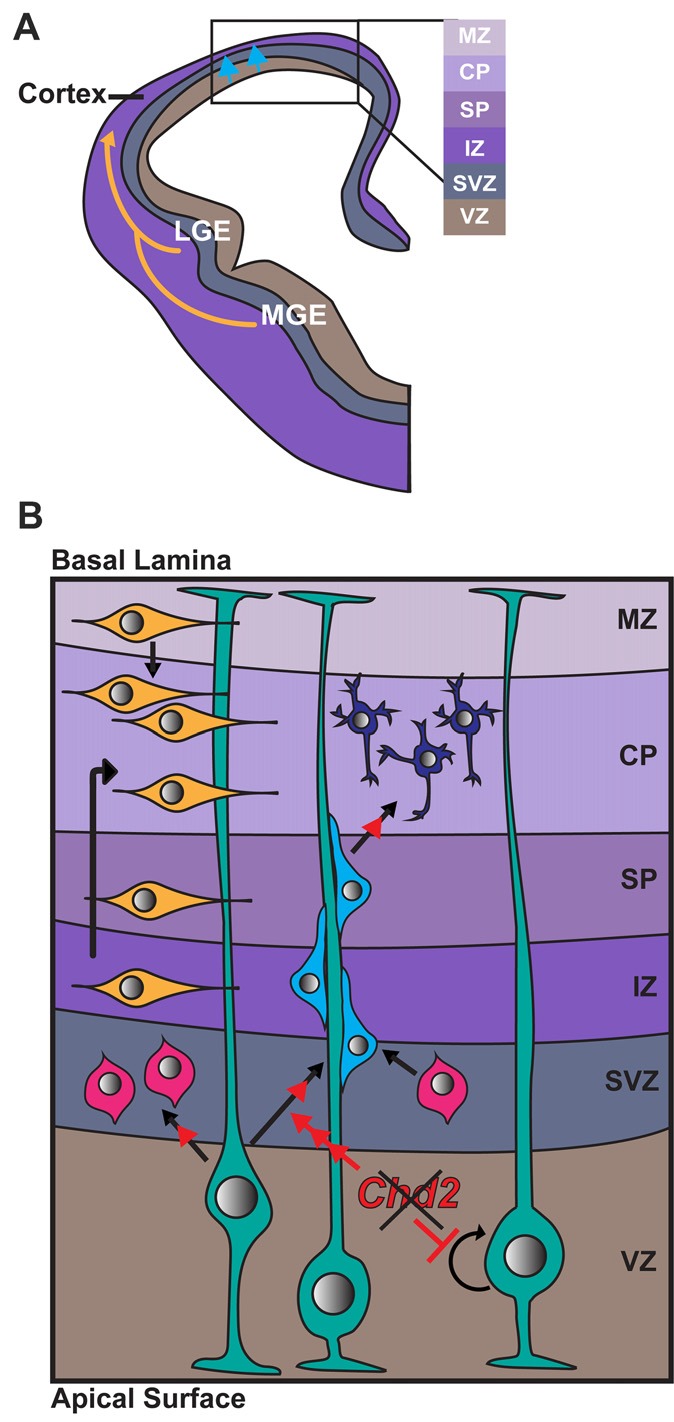
Role of CHD2 in differentiation of excitatory and inhibitory neurons. **(A)** A schematic representation of a coronal section of one half of an embryonic forebrain showing the subdivisions of the telencephalic proliferative zones: lateral ganglionic eminence (LGE) and medial ganglionic eminence (MGE). Excitatory neurons are born in the ventricular zone (VZ) of the cortex and migrate toward the brain surface (blue arrows). Most inhibitory interneurons of the cortex originate in the MGE and LGE, migrating tangentially to colonize the cortex (yellow arrows). Biallelic knockdown of *CHD2* *in vitro* has been shown to impair interneuron development. *In vivo*, CHD2 loss may hypothetically result in fewer interneurons migrating from the MGE/LGE or immature interneurons in the forebrain **(B)** Following migration to the cortex, GABAergic inhibitory interneurons (yellow) follow either a superficial migratory stream through the cortical plate (CP) and marginal zone (MZ) or migrate through the deep layers of the subventricular (SZ) and intermediate zones (IZ). In early stages of development, radial glial cells (RGCs; green) divide symmetrically to produce two new RGCs and replenish the progenitor pool (represented by circular arrow). As development progresses, Pax6+ RGCs begin to divide asymmetrically in the VZ, giving rise to excitatory neurons (shades of blue) or Tbr2+ intermediate progenitors (IPs; pink). Chd2 is expressed primarily in RGCs in the VZ/SVZ and is not expressed in IPs. When *Chd2* is disrupted, self-renewal of RGCs is diminished, there is an increase in the production of IPs, and more cells differentiate into glutamatergic neurons (red arrows; Anderson et al., 2001; Kriegstein and Noctor, 2004; Wu et al., 2011; Shen et al., 2015). CP, cortical plate; IZ, intermediate zone; LGE, lateral ganglionic eminence; MGE, medial ganglionic eminence; MZ, marginal zone; SP, subplate; SVZ, subventricular zone; VZ, ventricular zone.

The mammalian cerebral cortex is comprised of two major neuronal cell types, glutamatergic excitatory neurons and GABAergic inhibitory interneurons that are derived from distinct processes occurring during neurodevelopment. Cortical excitatory neurons are born in the ventricular and subventricular zones (VZ/SVZ) of the dorsal telencephalon (Figures 2A,B). Here radial glia cells divide either symmetrically to expand the progenitor pool, or asymmetrically to produce either a single radial glia cell along with an intermediate progenitor or neuron (Figure 2B; Paridaen and Huttner, 2014). Cortical inhibitory neurons are generated from distinct neuronal progenitor cells located primarily in the lateral ganglionic eminence (LGE), medial ganglionic eminence (MGE) and caudal ganglionic eminence (CGE) of the ventral forebrain. These neurons migrate tangentially into the dorsal forebrain and integrate in the cerebral cortex (Figure 2A). CHD2 has been shown to play a role in the development of both cortical excitatory and inhibitory neurons in mice *in vivo* and humans *in vitro*.

In mice, *Chd2* is predominantly expressed in Pax6+ radial glia in the VZ/SVZ from E12–E18 (Shen et al., 2015). Conversely, *Chd2* is rarely expressed in Tbr1+ intermediate progenitors (IPs) at the same embryonic stage. shRNA knockdown of *Chd2* by *in utero* electroporation during mouse embryogenesis leads to a reduction in the number of Pax6+ radial glia and increase in Tbr1+ IPs and Tuj1+ neurons. Overall these results suggest *Chd2* deficiency suppresses the self-renewal capacity of the radial glia and instead promotes premature neuronal differentiation (Shen et al., 2015). This premature differentiation likely leads to a rapid depletion of the progenitor pool, depletion of this pool can result in a smaller cortex and defects in later-born neurons (Homem et al., 2015). Indeed, patients with *CHD2* haploinsufficiency have reduced average head size and ~20% have microcephaly (Thomas et al., 2015; McRae et al., 2017).

CHD2 was recently demonstrated to play a crucial role in cortical inhibitory interneuron development *in vitro*. *CHD2* gene expression levels gradually increase during the differentiation of human embryonic stem cells (hESCs) to cortical interneurons by defined factors (Meganathan et al., 2017). CRISPR-Cas9 mediated biallelic knockout off *CHD2* resulted in fewer TUBB3 (TUJ1)+ neurons with shorter neurites, suggesting delayed or impaired differentiation. CHD2 knockout interneurons also exhibited electrophysiological defects that suggest hyper-excitability in these neurons (Meganathan et al., 2017). It is unclear how this increased excitability relates to reduced inhibitory synaptic input that is generally observed in epilepsy (Cherubini, 2012). Moreover, no electrophysiological defects were observed when these hESC CHD2 knockouts were differentiated to cortical excitatory neurons (Meganathan et al., 2017).

## CHD2: Role in Controlling Cellular Proliferation and Differentiation

Embryogenesis and development of an organism, and especially the brain, requires precise coordination of proliferation of progenitor cells and differentiation to specific terminally differentiated cells. Proliferation of precursors is achieved by progression through the cell cycle while differentiation is usually precipitated by a lengthening of the cell cycle in the G1 phase and transition to G0 phase and commitment to cell fate (Calegari et al., 2005). CHD2 has been implicated in both of these processes in multiple cell lineages, and its depletion has been shown to disrupt the balance between proliferation and differentiation.

As described above, *Chd2* siRNA-mediated knockdown in E13.5 mouse cortices leads to reduced proliferation in neural progenitor cells and premature differentiation of these progenitor cells *in vivo* (Shen et al., 2015). However, while hESCs lacking CHD2 resulted in delayed or impaired cortical interneuron differentiation, these hESCs did not exhibit reduced proliferation at the stem cell or early embryoid body stage, though later neural progenitor stages were not investigated for proliferative defects (Meganathan et al., 2017).

CHD2 has also been shown to play a role in controlling proliferation and differentiation in cell types other than neurons. *Chd2* knockdown by miRNA or siRNA in mouse myoblasts (C2C12s) results in differentiation defects, with decreased expression of differentiation-dependent myogenic genes and a lack of myotube formation (Harada et al., 2012). miRNA repression of *Chd2* did not affect cell cycle progression, however, indicating that proliferation was unaffected by *Chd2* knockdown. More recently this group found a similar differentiation defect in *Chd2* deficient mouse embryonic stem cells (mESCs; Semba et al., 2017). Depletion of Chd2 in mESCs resulted in ablation of differentiation potential and downregulation of markers for both myogenic and neural differentiation (Semba et al., 2017). As seen in C2C12 cells, there was no significant difference in proportion of cells in each stage of the cell cycle, indicating there were no proliferation defects in these cells (Semba et al., 2017).

Taken together, these studies have demonstrated differentiation defects in mouse ESCs, muscle and neuronal progenitor cells and human cortical interneurons. However, to date, only radial glia in mice have been shown to have defects in cellular proliferation *in vivo*. These discordant results indicate that CHD2 function may differ depending on tissue type and species and that CHD2 may have a unique role in neurogenesis of cortical excitatory neurons, distinct from its role in myogenesis and early development. As mentioned previously, upregulation of other CHD family members (thus compensation for CHD2 loss) may also play a role in cell-type and species-specific differences observed.

## CHD2: Role in Chromatin Remodeling and Gene Expression

**Figure 3:**
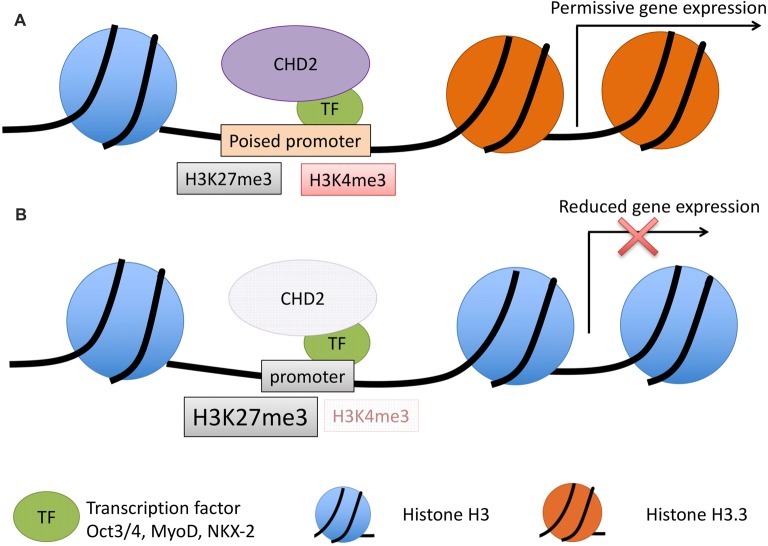
Model for CHD2 role in chromatin remodeling and epilepsy. **(A)** During development, CHD2 is recruited to poised promoters with the bivalent histone modifications, H3K27me3 (repressive) and H3K4me3 (activating) by interaction with specific transcription factors. CHD2 remodels chromatin at target genes by replacing histone H3 with H3.3 and creating a more permissive chromatin state whereby transcription of developmental genes can occur during differentiation. **(B)** When *CHD2* is mutated, promoters that would normally be poised for differentiation have an increase in the repressive H3K27me3 histone modification and H3.3 is not incorporated. These changes in the chromatin architecture restrict the expression of target genes during differentiation. During neuronal development this pathogenic mechanism likely leads to reduced expression of genes important in neuronal differentiation and impairments that ultimately lead to epilepsy and associated neurodevelopmental disorders.

CHD2 is a chromatin remodeler that has demonstrated ability to use the energy from ATP hydrolysis to remodel chromatin into periodic nucleosome arrays (Liu et al., 2015). The role of CHD2 in chromatin remodeling was first investigated in muscle cell differentiation. Co-immunoprecipitation (co-IP) experiments in C2C12 cells found that CHD2 interacts with H3.3, a histone variant incorporated into the nucleosome at transcriptionally active genes; while a mutant form of CHD2 lacking the chromodomain does not show any interaction with H3.3 (Harada et al., 2012). H3.3 is incorporated into myogenic gene promoters prior to their expression, but this incorporation is disrupted when *Chd2* is depleted by miRNA knockdown in C2C12s. CHD2 also showed an interaction with MyoD, a master regulator of skeletal muscle differentiation, and together MyoD and CHD2 bind to myogenic gene promoters. Suppression of *Chd2* expression in C2C12 cells decreased myogenic gene expression and halted myotube formation, indicating that *Chd2* is required for skeletal muscle differentiation. Combined, these data suggest a model where CHD2 is guided to differentiation-dependent genes by other transcription factors (e.g., MyoD in skeletal muscle) and that the chromodomain of CHD2 facilitates H3.3 incorporation into the nucleosome, poising genes necessary for differentiation for expression (Figure 3).

This model for transcription factor mediated recruitment of CHD2 is supported in other cell types in mouse and humans, including neurons. Co-IP experiments showed CHD2 associates with the transcription factor, OCT3/4 in mESCs, and ChIP-seq experiments demonstrate a significant overlap between CHD2 and OCT3/4 bound promoters, particularly at developmentally regulated genes (Semba et al., 2017). In human cortical interneurons, CHD2 ChIP-qPCR revealed an overlap with NKX2-1 binding at three candidate genes important for interneuron development, *ZIC1*, *SPRY1* and *PAX2*. Moreover, in *CHD2* depleted cells, overexpression of *NKX2-1* was insufficient to induce the expression of these target candidate genes, suggesting CHD2 is required to activate expression of neurodevelopmentally regulated genes (Semba et al., 2017). Collectively, these data suggest that CHD2 is recruited by cell type-specific transcription factors to developmentally regulated genes.

Multiple lines of additional evidence support the role of CHD2 in active gene expression. The Encyclopedia of DNA Elements (ENCODE) project profiled numerous histone modifications, transcription factors and chromatin remodelers using ChIP-seq to classify the genome into functional elements of the genome (Dunham et al., 2012). Reanalysis of the ENCODE data for CHD2 in three cell lines showed CHD2 is significantly enriched at active promoters (defined by H3K4me2/me3 and H3K9/27 acetylation) and enhancer regions (defined by H3k4me1/me2 and H3K9/27 acetylation; Siggens et al., 2015). These findings were replicated by histone modification mark profiling using ChIP-seq in mESCs, where, in addition to active promoters and enhancers, CHD2 was found at promoter regions in the bivalent state. These bivalent chromatin regions are characterized by both repressive (H3K27me3) and active (H3K4me3) histone modifications and are characteristic of developmentally regulated genes in stem cells. Gene ontology analyses of CHD2-bound promoters of active genes in mESCs revealed a significant enrichment of genes involved in chromosome organization, DNA repair, and chromatin modification, indicating that in addition to remodeling chromatin and recruiting transcription factors to target genes, CHD2 may also bind to and regulate other transcriptional regulators. The bivalent promoters bound by CHD2 in mESCs were enriched for genes involved in forebrain development, cell fate commitment, and central nervous system neuron differentiation, suggesting that CHD2 plays a role in directing mESCs toward neuronal lineages. CRISPR/Cas9 mediated depletion of *Chd2* in mESCs led to an increase in the repressive mark H3K27me3 at developmentally regulated bivalent genes, and this repression correlated with a decrease in expression of developmentally regulated, but not house-keeping or pluripotency related genes (Semba et al., 2017).

The CHD2-facilitated incorporation of H3.3 at target genes has also been extended to mESCs and human K562 cells (Siggens et al., 2015; Semba et al., 2017). In both cellular systems, knockdown of *Chd2*/*CHD2* led to a decrease in the levels of H3.3 incorporation. Moreover, as with muscle cells, the incorporation of H3.3 was specific to developmental genes in mESCs and *Chd2* loss correlated with reduced expression of these genes (Semba et al., 2017).

Collectively, these studies suggest CHD2 mediates the expression of developmentally regulated, bivalent or active genes during cellular differentiation by interaction with cell-specific transcription factors and by incorporation of H3.3 (Figure 3). In this way, CHD2 remodels chromatin into a permissive state, such that upon differentiation, developmentally regulated genes can be expressed. In human neurogenesis, CHD2 is likely to play a critical role in the remodeling of the chromatin state prior to neuronal differentiation, through interaction with NKX2-1 and other transcription factors that have yet to be identified.

## Other Biological Roles for CHD2

In addition to regulating gene expression, CHD2 also plays a role in the DNA damage response. Double strand break (DSB) repair through non-homologous end-joining (NHEJ) requires the expansion of chromatin, which is mediated by poly (ADP-ribose) polymerase (PARP) enzymes. PARP1 is able to sense DNA-damage, and when activated, forms poly ADP-ribose (PAR) chains that are able to act as a docking platform for other DNA repair factors (Wei and Yu, 2016).

PARP1 and CHD2 were found to interact through co-IP experiments where both proteins were overexpressed in osteosarcoma cell lines (U2OS cells). When DNA DSBs were inflicted by lasers in U2OS cells, CHD2 accumulated at sites of damage in a PARP1-dependent manner, indicating that PARP1 is required for recruitment of CHD2 to sites of DNA damage (Luijsterburg et al., 2016). The C-terminus of CHD2 (residues 1611–1828) was sufficient to mediate PAR-binding and accumulation at sites of DNA damage. CHD2 also played a role in the unfolding and expansion of chromatin, and the PAR-binding motif was sufficient to perform this function. Histone variant H3.3 is known to incorporate at sites of UV-induced DNA damage (Adam et al., 2013) and CHD2 was shown to contribute to H3.3 assembly at DSBs (Luijsterburg et al., 2016). These findings are in line with the ability of CHD2 to recruit H3.3 to regions of active transcription (Harada et al., 2012; Siggens et al., 2015). Through these experiments, a novel functional domain of CHD2 was identified. The C-terminal region containing residues 1611–1828 is a novel PAR binding domain and is required for proper DNA repair through NHEJ (Luijsterburg et al., 2016). The role of CHD2 in DNA-damage repair may explain why somatic variants in *CHD2* and other *CHD* family members are associated with multiple types of cancer (Thompson et al., 2003; Liu W. et al., 2012; Rodríguez et al., 2015; Lasorsa et al., 2016).

## CHD2 Dysfunction and Putative Pathogenic Mechanisms in Epilepsy

Epilepsy has long been regarded primarily as a channelopathy; where the underlying pathophysiology is due to a defect in ion channels, including the voltage-gated and ligand-gated channels. Unfortunately, despite extensive study of sodium channels and high-throughput screens by many pharmaceutical companies, few new therapies have been identified in the last decade. However, the unbiased nature of next generation DNA sequencing, and in particular exome sequencing, has revealed a role for a much broader range of biological functions in epilepsy. These include proteins that are chromatin remodelers, transcription factors, synaptic proteins, and cell signaling proteins, highlighting the diversity of pathogenic mechanisms implicated in epilepsy and moving the disorder beyond a channelopathy (Myers and Mefford, 2015). Understanding how these novel epilepsy genes cause this disorder represents a unique opportunity to not only identify new therapeutic targets, but also to gain insights into molecular neuroscience.

In this review, we have focused on the role of the chromatin remodeler CHD2 in neuronal development. Collectively, studies suggest that CHD2 is recruited to developmental genes by a transcription factor, where it remodels chromatin into a permissive state such that target genes can be transcribed upon differentiation (Figure 3). The target genes of CHD2 during neuronal development are not well-studied. Candidate ChIP-qPCR in inhibitory neurons showed CHD2 binding at the promoter regions for *ZIC1*, *SPRY1* and *PAX2*, and the expression of these genes was concomitantly reduced in CHD2 depleted cell lines. Moreover, *CHD2* knockdown in this inhibitory neuron model led to a reduced expression of genes involved in neurogenesis, synaptic transmission and genes involved in neurodevelopmental disorders, including epilepsy; upregulated genes were involved in cell adhesion and non-neural fate acquisition (Meganathan et al., 2017). Moreover, in mouse neuronal progenitor cells, candidate ChIP-seq revealed CHD2 binding at *REST* (RE1-silencing transcription factor) and that *REST* expression was decreased with *Chd2* loss. REST is a transcriptional repressor that prevents the expression of neuronal genes in non-neuronal cells. These results suggest that CHD2 binding at REST prevents its expression; this may be particularly important in the maintenance of proliferative state of neuronal progenitor cells, and this association may contribute to the premature neuronal differentiation observed with *Chd2* knockdown (Shen et al., 2015). REST has been previously implicated in epileptogenesis, though results are conflicting, with conditional REST loss being reported as both protective against, and predisposing to, seizures in different animal models (Hu et al., 2011; Liu M. et al., 2012). Collectively, these results suggest CHD2 regulates neurodevelopmental genes during differentiation in a manner similar to other cell-types and these defects are likely to at least in part, underpin the development of seizures in patients with *CHD2* haploinsufficiency.

*In vitro*, CHD2 interacts with the transcription factor NKX2-1 to control GABAergic inhibitory neuron development. Deficits in inhibitory neuron development and migration are an increasingly appreciated pathophysiologic mechanism in epilepsy. For instance, loss of function variants in the transcription factor *ARX* are associated with a myriad of X-chromosome linked epilepsies (Suri, 2005). *Arx* mouse models recapitulate these findings with multiple seizure types as well as impaired inhibitory neuronal migration from the MGE to the cortex and an accumulation of progenitors in the LGE and MGE (Colombo et al., 2007; Marsh et al., 2009). Moreover, various knockout models for *Nkx2-1*, as well as other transcription factors (*Dlx1/2*, *Dlx5/6*) that control GABAergic development, cause seizures in mice (Cobos et al., 2005; Butt et al., 2008; Wang et al., 2010). *CHD2* knockout resulted in impaired GABAergic inhibitory differentiation. However, perhaps somewhat counterintuitively, *CHD2* knockout interneurons also exhibited electrophysiological defects that suggest hyperexcitability. Impaired GABAergic interneuron differentiation is well established as pathogenic mechanism in epilepsy as described above. Meanwhile, hyperexcitability of these neurons, resulting in too much inhibition and seizures is more controversial; though promotion of neuronal synchronization or a disinhibition of epileptogenic networks resulting in unchecked excitatory networks are proposed pathogenic models (Klaassen et al., 2006; Frei et al., 2010). Moreover, neuronal maturity is a well-known factor that can affect stem cell derived neuronal electrophysiological properties (Isom, 2017). Overall, these studies illustrate impaired inhibitory interneuron differentiation and migration are an important pathophysiological mechanism in epilepsy, including *CHD2* haploinsufficiency, though further work is needed to understand pathophysiology at an electrophysiological level.

Hyperexcitability of excitatory neurons is also a well-known pathogenic mechanism in multiple cellular systems including heterologous expression systems, animal models, and stem cell derived neurons. For instance, duplications of *MECP2* causes early onset epilepsy and ID in males. Patient-derived induced pluripotent stem cells (iPSCs) from individuals with *MECP2* duplications differentiated to cortical excitatory cells showed increased dendritic arborization and increased synchronized activity of the neuronal network (Nageshappa et al., 2016). Conversely, CHD2 knockdown stem-cell derived excitatory neurons did not show any electrophysiological changes (Meganathan et al., 2017). However, *Chd2* knockdown *in vivo* resulted in reduced proliferation of radial glial cells and premature neuronal differentiation in the VZ/SVZ (Shen et al., 2015). These defects were similar to those seen in conditional *Arx* depletion mouse models, that have reduced proliferation of both radial glia and IPs, resulting in a reduced number or upper layer neurons (Marsh et al., 2009). Collectively, these results suggest impaired cortical excitatory cell development may be impaired with *CHD2* and *ARX* loss, both genes implicated in early onset epilepsies, though further studies are required to understand the putative pathogenic role in development of seizures.

One of the leading hypotheses for the initiation of seizures is an imbalance between excitatory and inhibitory synaptic inputs; the chronic imbalance between these two opposing systems likely plays a major role in the development of epilepsy. CHD2 has been shown to play a role in the development of cortical excitatory and inhibitory neurons in mice and humans, respectively. However, to date, the interaction of these two opposing inputs have not been investigated in a single model system of CHD2 loss.

## Future Perspectives

Although progress has been made deciphering the mechanism of CHD2 and its role in epilepsy in recent years, much is still unknown about the intricacies of chromatin remodeling in neuronal development and epilepsy. Improved animal models of *Chd2* depletion will be necessary in order to better understand these mechanisms. Epilepsy-associated pathogenic variants in *CHD2* follow a model of haploinsufficiency, therefore animal models are needed to recapitulate this model of pathogenesis. Current mouse and zebrafish models of *Chd2*/*chd2* depletion have utilized methods of gene disruption which do not ablate expression of the entire transcript. In the future, full knockout models, that rule out possible gain of function or dominant negative effects should be pursued. Appropriate controls are also important for such studies, such as utilizing multiple independent morpholinos targeting non-overlapping sequences of zebrafish *chd2* in order to confirm the phenotype (Bill et al., 2009; Blum et al., 2015). Rescue experiments reintroducing the wild type copy of *Chd2* will be an additional key control in mouse, zebrafish and stem cell studies. The advent of CRISPR/Cas9 technology may render the use of MOs obsolete, as a quick and efficient method of altering the zebrafish genome, although off-target effects remain a concern (Griffin et al., 2016). CRISPR/Cas9 could also be used to create patient-specific *CHD2* variants using a knock-in mouse model, which may re-capitulate human phenotypes more accurately. As there are DEE patients carrying pathogenic missense variants in different domains of CHD2, using knock-in models of these variants could uncover valuable information about the functions of the distinct domains. Ultimately, it will be necessary to generate animal models with conditional inactivation of *CHD2* in tissues of interest in order to delineate its function in distinct cell types. An alternative to mouse models is to utilize patient derived and genome edited stem cell models; these are being increasingly used to dissect the pathogenic mechanisms that underpin a variety of neurodevelopmental disorders, including epilepsy. As protocols improve to make a variety of excitatory and inhibitory neurons, as well organoids (or minibrains) these approaches will be more widely used and standards of use developed.

CHD2 has been found to regulate the chromatin architecture of developmentally important genes, poising these genes for expression during differentiation in various cell types. To date genome-wide CHD2 ChIP-seq and epigenomic profiling has not been performed in neuronal cell types to identify CHD2 target genes. In the future, these unbiased genome-wide experiments should be pursued using stem cell modeling in patient-derived and genome-edited cells, to identify CHD2 targets as well as alterations to the chromatin architecture. These studies may illuminate potential targets for therapeutic interventions. Moreover, to date CHD2 has been found to play a role in inhibitory neurons derived from hESCs and cortical excitatory neurons in mice. In the future, it will be key to study these two opposing neuronal systems at both a cell autonomous and network level. Typically, these experiments have been performed in knockout, or conditional knockout, mice. However, the use of patient-derived organoids is also an exciting area of burgeoning research, including the development or both ventral and dorsal organoids, and fusion of these two subtypes to study GABAergic migration *in vitro* (Birey et al., 2017).

Additional research is required to decipher the effects of CHD2 on proliferation, and why only some CHD2-deficient cell types show proliferation defects. This could be due to distinct expression patterns of *CHD2* and its potential binding partners in different cell types. The role of CHD2 in DNA-repair likely is linked to its effects on proliferation, and the interplay between DNA-repair, cell cycle exit, and the balance between proliferation and differentiation during neurogenesis should be explored. *Chd2* deficient mice have a reduction in their ability to repair DSBs, leading to increased cell death. Whether this defect leads to the decreased proliferation and increased differentiation seen in neuronal models remains to be seen.

Finally, while *CHD2* haploinsufficiency is a rare cause of genetic epilepsy, many of the pathogenic mechanisms, particularly during neuronal development are likely to be overlapping with other genetic epilepsies. Indeed, preliminary transcriptome studies suggest CHD2 may be an upstream regulator of other genes implicated in epilepsy and that novel therapeutics may be applicable to broader range of patients with these disorders. In addition, there is extensive overlap between risk genes for autism and cancer, including *CHD2* and other chromatin remodelers, *CHD7*, *CHD8*, *ARID1B* and *ATRX* (Crawley et al., 2016). If we can better understand the common pathways between cancer and neurodevelopmental disorders, this could lead to breakthroughs in drug development. Previously developed cancer drugs that share common targets with neurological disorders could be repurposed for new use, and vice versa. HDAC inhibitors are an example of therapeutics that have applications in various diseases, including cancer, neurodegenerative disorders and neurodevelopmental disorders (Qiu et al., 2017); there are likely other such targets involved in chromatin remodeling pathways yet to be discovered.

## Author Contributions

K-ML and GC contributed equally to the writing of this manuscript.

## Conflict of Interest Statement

The authors declare that the research was conducted in the absence of any commercial or financial relationships that could be construed as a potential conflict of interest.
